# Characterizing Demographic and Geographical Differences in Health Beliefs and Dietary Habits Related to Colon Cancer Risk in US Adults

**DOI:** 10.3389/fnut.2020.568643

**Published:** 2020-10-07

**Authors:** Megan N. Schaberg, Kristen S. Smith, Michael W. Greene, Andrew D. Frugé

**Affiliations:** Department of Nutrition, Dietetics, and Hospitality Management, Auburn University, Auburn, AL, United States

**Keywords:** colon cancer, diet, health belief model, red meat, green leafy vegetables, health behavior

## Abstract

**Background:** Colon cancer (CC) risk is increased by behavioral factors including a diet high in red meat (RM) and processed meat; excess adiposity has contributed to a rise in CC in younger adults. The willingness of at-risk adults to modify behaviors to reduce CC risk warrants further investigation.

**Methods:** The previously validated Dietary Habits and Colon Cancer Beliefs Survey (DHCCBS) was used to assess attitudes and beliefs related to CC risk and diet behavior. An abbreviated food frequency questionnaire was included in the survey to quantify RM and green leafy vegetable (GLV) intake over the previous 30 days. Independent samples *t*-tests compared RM and GLV intake and DHCCBS responses. One-way analysis of variance with *post-hoc* LSD correction was completed to assess these differences within three age groups (<35, 35–44, and 45–54 years old) and between U.S. Census Bureau geographical regions.

**Results:** Eight hundred and thirty eight survey responses were analyzed. Perceived severity of CC diagnosis was significantly lower in younger adults (<35) compared to older adults (35–44, *p* = 0.042; 45–54, *p* = 0.003). Furthermore, younger adults (<35) perceived fewer barriers (i.e., taste preference) to GLV consumption than their older adult counterparts (35–44, *p* = 0.019; 45–54, *p* = 0.002). Few regional differences in habitual RM consumption were observed, however, several disparities were observed with GLV.

**Conclusion:** These findings from the DHCCBS indicate health beliefs toward CC risk are influenced by an individual's age and dietary habits. Additionally, regional differences in GLV consumption indicate opportunities for risk-reduction-focused health messages, particularly in the southern United States where CC incidence and mortality are highest.

## Introduction

Colon cancer (CC) is the third most common cancer in men and women in the United States (US), and the third leading cause of cancer deaths (1). Both hereditary and environmental factors contribute to CC risk, such that excess adiposity in adolescence confers 28% greater lifetime risk for CC in women (2). Increased waist circumference in adulthood alone is associated with a 53% increased risk of CC (3). Though obesity is a multifactorial disease, diet is a major contributor to CC risk. In the US, ~38.3% of new CC cases in 2015 were directly attributed to suboptimal diet (4). The Western diet, rich in red meat (RM) and processed foods and lacking in vegetables, is heavily implicated in CC development (5), with a recent meta-analysis indicating a 30% increased risk of CC for adults consuming this dietary pattern (6).

Heme, the iron-carrying molecule conferring color to RM, readily oxidizes lipids, and other molecules in the lumen of the colon, causing cytotoxicity and epithelial proliferation in the gut, which can promote carcinogenesis (7). Heme is composed of a porphyrin ring surrounding iron which is easily oxidized and absorbed primarily in the large intestine (8). When red or processed meats are consumed in excess, heme accumulates within the colon, contributing to cytotoxic effects (9). Furthermore, bacteria residing in the lumen interact with heme, producing toxic metabolites to further increase damage (10). Additionally, different cooking methods create varying amounts of mutagenic heterocyclic amines (11), although the effects of cooking methods on RM related CC risk is still being investigated in humans.

In contrast, diets with high green leafy vegetable (GLV) intake are associated with a decreased risk of CC (12). GLV are high in chlorophyll, fiber, and flavonoids and carotenoids, all of which are known for their anti-cancer effects. Chlorophyll, a structural analog to the porphyrin ring of heme, prevents mucosal damage, and hyperproliferation effects by competing with and binding to heme molecules (13). Fiber increases bowel motility and bacterial fermentation, decreasing the concentration of intestinal carcinogens (14). Flavonoids and carotenoids, which are abundant in GLVs, are associated with lowered risk of CC (15) and can reduce oxidative stress, increase apoptosis, and inhibit cell proliferation (16, 17).

Recommendations from the American Institute of Cancer Research (AICR) to reduce CC risk include behavioral changes in modifiable risk factors. These include consuming 5 servings of vegetables daily, with emphasis on 1 serving derived from GLV (18). The AICR also recommends reducing RM intake to 70 g per day or less. It has been observed that increased GLV consumption may be protective against CC in RM-rich diets (19), but disparities in dietary patterns, and health-seeking behaviors may confound epidemiological diet-related observations (20, 21). Finally, the American Cancer Society suggests CC screenings starting at the age of 45 with no known family history of CC (22). CC screenings are associated with a reduction in CC mortality; however, approximately half of the US population aged 50 and older do not comply with recommended CC screenings (23).

Awareness of risk factors associated with CC is important for reducing behaviors that could lead to CC development. However, knowledge of risk factors may not be sufficient to stimulate behavioral change (24). Western countries have been associated with an unwillingness of adults to adjust dietary patterns, regardless of the health outcome (25). Nonetheless, understanding the benefits of engaging in a health behavior can influence whether an individual will seek changes.

The Health Belief Model (HBM) utilizes five domains: perceived susceptibility, perceived severity, perceived benefit, perceived barriers, and cues to action, to examine health-related behaviors (24). This behavioral model has been used for almost seven decades; in recent years is has been used to understand behaviors ranging from the adherence of treatment for Tuberculosis, to the self-care attitudes of diabetics, and behaviors associated with cervical cancer risk (26–28). The Dietary Habits and Colon Cancer Beliefs Survey (DHCCBS) was developed and validated using the HBM to assess beliefs and attitudes related to diet and CC risk (29). Herein, we further analyzed associations between DHCCBS responses and dietary intake, within different age groups and US regions to explore the relationship between dietary habits and expected health outcomes in relation to CC risk.

## Methods

Auburn University's Institutional Review Board (IRB) granted approval for this study. Prior to starting the survey, participants were notified about confidentiality and their right to discontinue at any time. After reading the IRB information, consent was inferred via initiation of the survey. Participants were enlisted in May 2018 through the online portal Amazon Mechanical Turk (mTurk) to access the survey instrument. Compensation was given upon survey completion and validation of responses.

Methods of development and validation of the DHCCBS are published and can be readily accessed for further details (29). Briefly, 13 HBM Likert scale questions assessed the five domains of health behavior related to CC: one susceptibility question, two severity questions, two barrier questions, three benefits questions, and four cues-to-action questions. Twenty questions from the previously validated Dietary Health Questionnaire II (DHQII) quantified consumption of GLV and total RM (including beef, pork, lamb, and processed meats) over the previous 30 days (30). Three additional questions were added from the previously validated Meat Module Questionnaire (MMQ) to assess exposure to carcinogens in burgers, bacon, and steaks (31). Two attention check questions were included to validate survey responses and 8 questions assessed demographic and anthropometric information (age, sex, race, education, height and weight, and region). A goal sample size of 1,000 was determined using the rule of thumb: 10:1 subject to variable ratio (32). Serving sizes were estimated using frequency of intake over the last 30 days, as determined from DHQII responses, and multiplied by respective daily frequencies of consumption. Average weekly consumption amounts were converted into cooked-cup equivalents for GLV and ounce equivalents for RM and used to calculate total servings per day. One half cup of GLV and 2.5 ounces of RM were used as standards for servings of each respective food group. Methods for scoring the instrument were previously reported (29). Regional information was collected via zip code of respondents and characterized within the 4 regions of the US, according to the Census Bureau (33).

Exploratory analyses were conducted to evaluate relationships between DHCCBS questions and RM and GLV intake between age groups and regions. RM and GLV intake and DHCCBS responses were compared using independent sample *t-*tests. One-way analysis of variance (ANOVA) for each age group was used to compare individual barrier questions and RM and GLV consumption. *Post-hoc* analyses between barrier question responses were evaluated with LSD correction for multiple comparisons. Scores from the four questions within the cues to action domain were totaled together to generate one variable representing total domain score. Multivariable linear regression models were used to evaluate effectiveness of demographic predictors (i.e., sex, race, education, BMI category, and geographical region) on total scores from cues to action domain. Simple linear regression models were used to evaluate associated between DHCCBS questions on reported habitual GLV and RM servings per day within this subset of participants (*n* = 838). Responses were grouped into either disagree (including strongly disagree, disagree, and neither agree nor disagree responses) or agree (including strongly agree and agree responses), with disagree as the reference group for the models. Results were considered significant with a *p* ≤ 0.05.

## Results

A total of 838 respondents were aged 54 years or less and included in the analysis. Respondents included 48.8% males, 77.7% white, and 54.4% had at least a bachelor's degree (Table 1). Males were overrepresented in the youngest age group and white adults were overrepresented in the oldest age group.

**Table 1:** Characteristics of dietary habits and colon cancer beliefs survey respondents.

	**Total**	**<35**	**35–44**	**45–54**	**Between**
	**(*n =* 838)**	**(*n =* 487)**	**(*n =* 227)**	**(*n =* 124)**	**group**
					***p*-value**
GLV svgs/day Mean (SD)^*^	1.00 (1.15)	1.04 (1.18)	0.99 (1.09)	0.90 (1.16)	0.525
RM svgs/day Mean (SD)^*^	0.93 (0.94)	0.96 (0.98)	0.93 (0.97)	0.81 (0.66)	0.494
**Sex N (%)**					0.001
Female	429 (51.2)	223 (45.8)	132 (58.1)	74 (59.7)	
Male	409 (48.8)	264 (54.2)	95 (41.9)	50 (40.3)	
**Race** ***N*** **(%)**					0.008
Asian	84 (10)	63 (12.9)	19 (8.4)	2 (1.6)	
Native American	10 (1.2)	7 (1.4)	1 (0.4)	2 (1.6)	
Black	49 (5.8)	33 (6.8)	11 (4.8)	5 (4)	
Pacific Islander	2 (0.2)	1 (0.2)	1 (0.4)	NA	
White	651 (77.7)	359 (73.7)	180 (79.3)	112 (90.3)	
More than one race	42 (5)	24 (4.9)	15 (6.6)	3 (2.4)	
**Education** ***N*** **(%)**					0.296
< High School	4 (0.5)	3 (0.6)	NA	1 (0.8)	
HS Grad/GED	81 (9.7)	51 (10.5)	17 (7.5)	13 (10.5)	
Some College	209 (24.9)	128 (26.3)	49 (21.6)	32 (25.8)	
Associate's Degree	85 (10.1)	42 (8.6)	30 (13.2)	13 (10.5)	
Bachelor's Degree	343 (40.9)	198 (40.7)	102 (44.9)	43 (34.7)	
Master's Degree	92 (11)	48 (9.9)	25 (11)	19 (15.3)	
Professional Degree	17 (2)	13 (2.7)	3 (1.3)	1 (0.8)	
Doctorate	7 (0.8)	4 (0.8)	1 (0.4)	2 (1.6)	
**BMI category** ***N*** **(%)**					0.116
Underweight	25 (3)	17 (3.5)	5 (2.2)	3 (2.4)	
Normal weight	389 (46.4)	238 (48.9)	103 (45.4)	48 (38.7)	
Overweight	259 (30.9)	151 (31)	64 (28.2)	44 (35.5)	
Obese	165 (19.7)	81 (16.6)	55 (24.2)	29 (23.4)	
**Region** ***N*** **(%)**					0.523
Northeast	148 (17.7)	95 (19.5)	32 (14.1)	21 (16.9)	
Midwest	163 (19.5)	94 (19.3)	43 (18.9)	26 (21)	
South	284 (33.9)	157 (32.2)	82 (36.1)	45 (36.3)	
West	212 (25.3)	123 (25.3)	63 (27.8)	26 (21)	

* *Amounts presented as servings per day, (1 serving = 2.5 ounces RM and 0.5 cooked cup equivalents GLV)*.

Relationships within age groups and DHCCBS responses were further explored in Table 2. Perceived quality of life-related severity of CC was significantly lower in younger adults (<35) compared to older adult cohorts (35–44, *p* = 0.042; 45–54, *p* = 0.003). Additionally, older participants (45–54) reported greater perceived benefits of increasing GLV consumption to reduce CC risk than the younger age group (<35; *p* = 0.006). Interestingly, younger participants (<35) received more recommendations from friends and family members to increase GLV intake in order to reduce CC risk (35–44, *p* = 0.033; 45–55, *p* = 0.002).

**Table 2:** Dietary habits and colon cancer beliefs survey responses of US men and women.

	**Total**	**<35**	**35–44**	**45–54**	
	**Mean (SD)**	**Mean (SD)**	**Mean (SD)**	**Mean (SD)**	**Between group *p-*value**
**Susceptibility**					
Please rate your perceived risk for developing colon cancer in your lifetime	2.12 (0.60)	2.08 (0.63)	2.18 (0.56)^*^	2.17 (0.58)	0.068
**Severity**					
Colon cancer can severely decrease my quality of life	4.67 (0.77)^§^	4.61 (0.85)^†^^#^	4.74 (0.67)^*^	4.77 (0.59)^*^	0.046
Colon cancer could lead to death	4.7 (0.70)^§^	4.65 (0.79)^†^	4.78 (0.57)^*^	4.78 (0.50)	0.028
**Perceived benefits**					
If I eat less red meat I could decrease my risk of developing colon cancer	3.76 (0.97)	3.69 (0.952)	3.84 (0.96)	3.88 (1.03)	0.053
If I eat more green leafy vegetables I could decrease my risk of developing colon cancer	4.14 (0.85)^§^	4.09 (0.85)^#^	4.15 (0.85)	4.32 (0.81)^*^	0.024
**Perceived barriers**					
I don't like the taste of other protein-rich foods	2.08 (1.00)	2.09 (0.98)	2.06 (1.08)	2.07 (0.94)	0.916
I don't like the taste of green leafy vegetables	1.95 (1.16)^§^	2.06 (1.19)^†^^#^	1.85 (1.16)^*^	1.71 (0.94)^*^	0.003
I can't imagine never eating red meat	3.25 (1.53)	3.26 (1.53)	3.3 (1.55)	3.14 (1.48)	0.628
**Cues to action**					
A healthcare provider has recommended that I eat less red meat	1.64 (0.99)	1.63 (0.96)	1.62 (1.01)	1.72 (1.09)	0.627
A friend or family member has recommended that I eat less red meat	1.84 (1.18)	1.92 (1.22)^†^	1.72 (1.14)^*^	1.77 (1.10)	0.085
A healthcare provider has recommended that I eat more green leafy vegetables	2.69 (1.46)	2.71 (1.44)	2.68 (1.49)	2.61 (1.52)	0.798
A friend or family member has recommended that I eat more green leafy vegetables	2.78 (1.49)^§^	2.91 (1.46)^†^^#^	2.66 (1.52)^*^	2.44 (1.46)^*^	0.003

§ *Between group significance*;

* *significance between <35 age group*;

† *significance between 35–44 age group*;

# *significance between 45–54 age group*.

**Figure 1:**
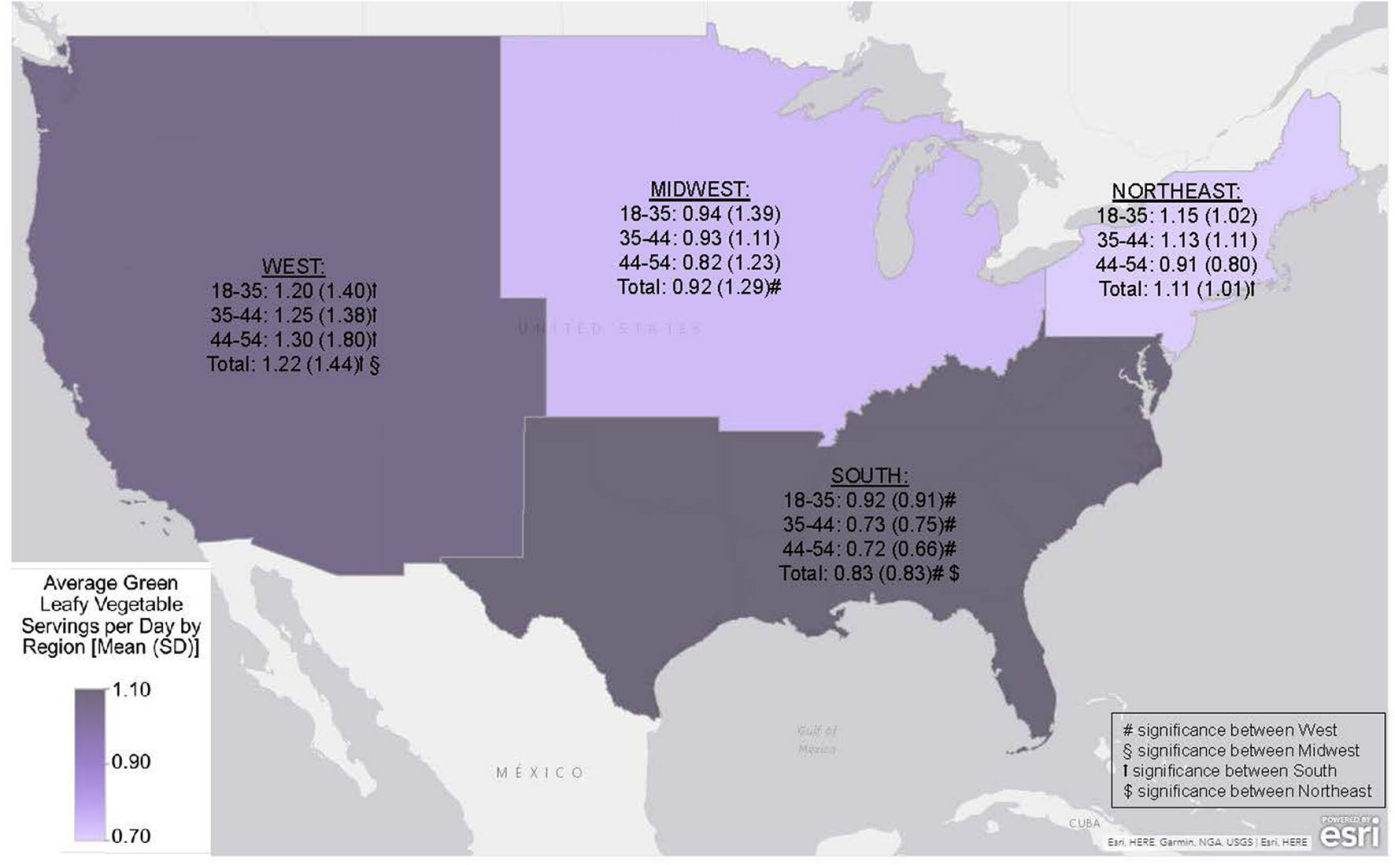
Average green leafy vegetables (GLV) consumption within US Census Regions by age group (1 serving = 0.5 cooked cup equivalent of GLV) Data is presented as mean (SD) and differences considered significant at *p* < 0.050.

**Figure 2:**
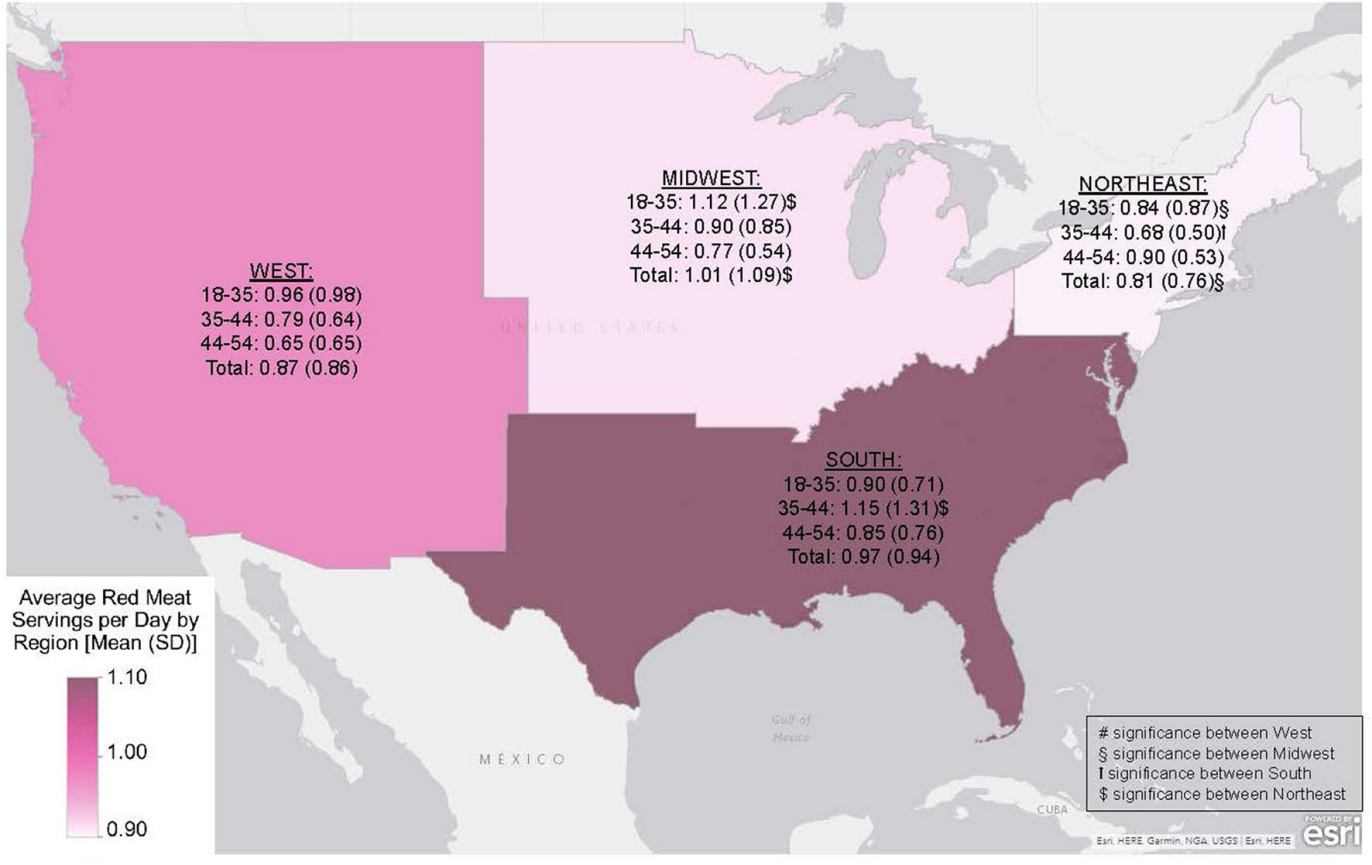
Average red meat (RM) serving per day within US Census Regions by age group, presented as mean (SD) (1 serving = 2.5 ounces RM). Differences considered significant at *p* < 0.05.

GLV and RM intake were compared between US regions within these age groups to further understand these relationships. The middle age group (35–44) in the Southern US consumed significantly more RM than corresponding individuals in the Northeastern region (*p* = 0.021). Furthermore, each age group differed in GLV consumption between the South and West regions (<35, *p* = 0.050; 35–44, *p* = 0.005; 45–54, *p* = 0.044; total, *p* < 0.001). Figures 1, 2 compare the different age groups and consumption of GLV and RM, respectively, within each region.

Several characteristics significantly predicted cues to action scores (Table 3). Predicted total scores of Asian (3.3 ± 0.5) and Native American (3.0 ± 1.2) respondents were over three points higher than white respondents (*p* < 0.001; *p* = 0.015, respectively); and females were approximately one point lower than males (*p* < 0.001). Additionally, overweight and obese respondents had higher predicted scores than normal weight respondents (*p* = 0.035; *p* = 0.001, respectively). Education and geographical location were not significant predictors of total cues to action scores.

**Table 3:** Multivariable linear regression analysis evaluating predicted Cues to Action scores between demographic groups of US adults completing the dietary habits and colon cancer beliefs survey.

**Variable**		**Predicted total scores^#^**	**ß**	**SE**	*p*-value^*^
**Sex**	Male	9.5	Ref		
	Female	8.5	−0.988	0.276	**<0.001**
**Race**	White	8.5	Ref		
	Asian	11.7	3.248	0.451	**<0.001**
	Native American	11.5	3.022	1.240	**0.015**
	Black	10.0	1.522	0.577	**0.008**
	Pacific Islander	5.5	−2.978	2.757	0.280
	Mixed	9.0	0.498	0.620	0.421
**Education**	Bachelor's Degree	9.1	Ref		
	< High School	8.3	−0.800	2.025	0.693
	HS Grad/GED	8.9	−0.124	0.497	0.804
	Some College	8.5	−0.581	0.353	0.101
	Associate's Degree	9.5	0.433	0.488	0.375
	Master's Degree	9.2	0.135	0.473	0.775
	Professional Degree	8.7	−0.344	1.000	0.731
	Doctorate	9.7	0.665	1.537	0.666
**BMI category**	Normal Weight	8.5	Ref		
	Underweight	9.6	1.137	0.825	0.168
	Overweight	9.1	0.676	0.321	**0.035**
	Obese	9.7	1.222	0.372	**0.001**
**Geographical region**	South	9.0	Ref		
	Northeast	9.1	0.102	0.401	0.800
	Midwest	8.6	−0.436	0.388	0.262
	West	9.0	0.027	0.358	0.941

* *Significant p-values are indicated in bold (p = <0.05)*;

# *total predicted scores within Cues to Action domain as calculated using standard regression equation*.

Individuals who perceived high CC susceptibility were predicted to consume more GLV (0.4 ± 0.1) and less RM (−0.3 ± 0.1) compared to those with lower perceived CC susceptibility (*p* < 0.001). Additionally, those who perceived benefits of GLV were indeed predicted to consume more GLV (−2.5 ± 1.1) compared to those who did not perceive benefits from GLV consumption (*p* = 0.01). Furthermore, predicted RM consumption was higher in individuals who perceived barriers to eliminating RM intake (−0.6 ± 1.2; *p* < 0.001). Supplementary Table 1 reports associations between reported habitual GLV and RM intake with DHCCBS questions.

## Discussion

These results suggest there are age-related disparities in health beliefs related to diet and CC, as well as regional differences in GLV and RM consumption. Specifically, GLV intake varied greatly between the southern and western US across all age groups. Survey respondents reported consuming almost twice the amount of RM compared to previous reports of 2015–2016 NHANES data (34). This is likely because we used FFQ to assess habitual intake as opposed to 24-h dietary recalls in NHANES and we included red processed meats in this value. Referencing NHANES 2015–2016 nutrient intake tables, mean daily Vitamin K consumption of 120.9 μg would reflect ~1/8 cup GLV, which is similar to our observed 1 cup GLV per week (35).

Diet, as well as other biological and environmental factors, contribute to CC risk (36). Lagerlund et al. (37) estimate that approximately one third of cancers in the developing world could be prevented by addressing known modifiable risk factors. One study suggests providing health-related knowledge about the effects of meat intake is not sufficient to reduce meat consumption due to individuals' mistrust of information sources (38). This recent systematic review concluded that individuals do not consider reducing intake of specific foods to improve health; rather, they contemplate increasing intake of fruits and vegetables (38). Similarly, while individuals may be aware of this relationship between dietary habits and cancer risk, many lack the knowledge of which specific foods or nutrients influence this risk (39). A study by Sullivan et al. (40) concluded that individuals were more likely to change health-related behaviors when they believed their actions could influence a health-related outcome, regardless of their perceived risk for that outcome. Additionally, when surveyed about cancer prevention strategies, a majority of respondents did not consider nutrition as a prevention strategy (40). Moreover, an intervention providing personalized healthcare screenings tailored to individuals' gender and bodyweight status risks resulted in positive changes of unhealthy behaviors, with individuals increasing daily intake of fruits and vegetables (41). Aligning with current research, healthcare recommendations could have better success in behavioral change if the focus shifted from decreasing RM to increasing GLV intake. Healthcare interventions and recommendations focused on enhancing patients' knowledge of nutrition and corresponding risk could improve strategies for diminishing CC risk and increase adherence to these health-related behaviors.

Recent trends in CC epidemiology indicate a shift in what was once considered a rare cancer that only happened in older adults, and is now occurring increasingly in young adults (1). For both genders over the age of fifty, the incidence of CC has decreased (42). However, in a retrospective cohort study, the predicted incidence of CC by the year 2030 will increase by 90.0% for adults ages 20–34 (43). Our results indicate the younger participants do not recognize the severity of CC diagnosis compared to older participants; thus, younger adults at increased risk of CC due to lifestyle factors may benefit most from tailored public health messages.

Regression analyses indicate respondent characteristics predict cues to action scores. Certain races (Asian, Native American, and black) could benefit from more recommendations to modify behaviors related to CC risk. Incidence of CC in Native Americans (43.3%) and non-Hispanic blacks (45.7%) is higher compared to non-Hispanic whites (38.6%), suggesting these recommendations are not misplaced (1). Moreover, increased BMI is associated with increased CC risk (44), corresponding to our report of overweight and obese respondents with higher predicted scores in cues to action domain. While CC risk is associated with obesity, dietary interventions of fruits and vegetables may provide protective effects against obesity-related CC through altered gene expression (45).

While this study provides insight into health behaviors and attitudes relative to CC, it is not without limitations. The population of this study was disproportionate to the American population, as most of the participants were from the Southeastern US. Similarly, African Americans were underrepresented. Compared to the national average of about one-third of Americans, more than half of the participants herein earned at least a Bachelor's Degree (46). Furthermore, socioeconomic status and area of educational background was not assessed within the survey instrument, although these factors can influence dietary habits and health-related knowledge (47, 48). Moreover, assessment of nutrition-related literacy could have provided important insight to improving current public health strategies. Additionally, the gut microbiome is known to play an important role in the pathogenesis of CC (49), however, analysis of the microbiome was not within the scope of this study, and therefore limits our understanding of the relationship between diet and CC-related health behaviors. Finally, as with any online survey instrument, there is chance for inaccurate reports of information or disclosures from participants.

These DHCCBS results suggest dietary habits influence the willingness to change health-related behaviors. Participants with the greatest risk of CC are unaware of their risk and are less likely to make the necessary changes to improve their health outcomes. Public health recommendations should provide feasible health behaviors and consider regional differences in dietary patterns. Providing CC screenings for younger adults that also include behavioral risk reduction guidance may decrease CC morbidity and mortality. Dietary recommendations that address dietary habits and behavioral barriers, such as increasing GLV over reduction of RM to obtain benefits may be most beneficial.

## Data Availability Statement

The raw data supporting the conclusions of this article will be made available by the authors, without undue reservation.

## Ethics Statement

The studies involving human participants were reviewed and approved by Auburn University Institutional Review Board. The patients/participants provided their written informed consent to participate in this study.

## Author Contributions

AF, MG, and KS designed the original study. AF obtained funding for the original study. MS, AF, and KS conducted analyses and drafted the original manuscript. All authors provided critical feedback to the final manuscript.

## Conflict of Interest

The authors declare that the research was conducted in the absence of any commercial or financial relationships that could be construed as a potential conflict of interest.
